# A Standardised Protocol for Pre-operative Pelvic Radiographs for Templating in Total Hip Arthroplasty

**DOI:** 10.7759/cureus.50687

**Published:** 2023-12-17

**Authors:** Abdus S Wasim, Muaaz Tahir, Ali Ridha, Amil Sinha, Shakir Hussain

**Affiliations:** 1 Orthopaedics and Trauma, Sandwell and West Birmingham NHS Trust, Birmingham, GBR; 2 Trauma and Orthopaedics, Royal Orthopaedic Hospital, Birmingham, GBR; 3 Orthopaedics, Wythenshawe Hospital, Birmingham, GBR

**Keywords:** hip arthritis, hips, orthopaedics & traumatology, musculoskeletal radiology, general radiology, preoperative templating, total hip arthroplasty: tha

## Abstract

Purpose: Digital templating using pre-operative radiographs enables pre-operative planning for total hip arthroplasty (THA). This allows surgeons to reproduce hip biomechanics effectively, reducing the risk of post-operative complications. Pelvic radiographs demonstrating the head, neck, trochanters, and proximal one-third of the femoral shaft allow calculation of key measurements including femoral offset and limb length discrepancy (LLD). Currently, no standardised guideline exists for obtaining pre-operative radiographs for templating in THA.

Materials and methods: A single-blinded retrospective cohort study assessing the quality of pre- and post-operative radiographs of 195 patients who underwent elective THA for osteoarthritis over a two-year period was performed. Quality was rated as good, fair or poor, respectively, depending upon whether ≥2, 1 or none of the following were met: Pubic symphysis (PS) and coccyx in a straight line with 1-3 cm between the superior edge of the PS and tip of coccyx, trochanters distinguishable, obturator rings symmetric. Post-operative images were assessed to determine whether the distal end of the implanted prosthesis was visible.

Results: The sample consisted of 195 patients. Pre-operatively 115 (59%) radiographs were classified as good, 71 (36.4%) fair and 9 (4.6%) poor. Post-operatively 46 (23.6%) were classified as good, 114 (58.4%) as fair and 30 (15.4%) as poor. In the post-operative radiographs, 25.6% did not include the distal tip of the prosthesis.

Conclusion: This study highlights significant scope to improve the quality of pre-operative radiographs, allowing accurate templating to optimise outcomes for THA. A protocol is recommended whereby the pelvic radiograph is centred on the PS at the lesser trochanter level, ensuring adequate exposure of the proximal femur, acetabulum and iliac crests.

## Introduction

Digital templating of pre-operative radiographs is increasingly utilised by surgeons pre-operatively to plan for total hip arthroplasty (THA), aiming to minimise post-operative complications, including risks of periprosthetic fracture, dislocation and loosening which can result from inappropriate prosthesis sizing and positioning [1]. Templating can help to predict the implant sizes and positioning required for restoration of normal hip biomechanics; specifically, the limb length and femoral offset. Limb length discrepancy (LLD) is the most common cause of patient dissatisfaction and litigation after THA [2,3]. Femoral offset, which refers to the perpendicular distance from the centre of rotation of the hip joint to the longitudinal axis of the femoral shaft, is an equally important measurement as it determines the moment arm of the abductor muscles as well as the wear and stability of the implants. Thus, preserving or restoring these parameters leads to better abductor muscle function and improves the longevity of the implants [4]. The shift towards biological (cementless) fixation of femoral implants also mandates a better appreciation of the osseous anatomy in order to minimise the risk of intraoperative fracture or early implant loosening. 

The accuracy of pre-operative templating relies heavily upon the quality of the radiographs being used to perform these measurements. The standard anteroposterior (AP) radiographs of the pelvis that are routinely used in clinical practice are generally centred on the sacrum and thus do not capture the proximal femur adequately. Similarly, an AP view of the affected hip alone is also inadequate as it prevents comparison with the contralateral side. The ideal radiograph for pre-operative planning would be an AP view of the pelvis centred over the pubic symphysis (PS) obtained with the patient lying supine with the hips in 10° to 15° of internal rotation [5]. This allows a true AP view of the femoral neck, which has a normal anteversion of 10° to 15°. Varying internal rotation of the femur may underestimate the femoral neck length and the femoral offset in radiographs. Theoretically, if the anatomy of the pathological hip is well preserved, templating can be performed on the same side. However, if the pathological hip is deformed or radiographed in an inappropriate position, the healthy contralateral hip can be templated, and the result is mirrored to the pathological side. Conventionally, limb length discrepancy is determined by measuring the distance from the antero-superior iliac spine and the medial malleolus on a full-length antero-posterior standing radiograph of both lower limbs [5]. Although a full-length antero-posterior standing radiograph of both lower limbs from the iliac crest to the tibiotalar joint should ideally be obtained, this seldom is the case. It is well recognised that standard AP radiographic views of the pelvis pre-operatively are helpful in assessing LLD in THA by various measurements (using the bi-ischial line or radiological teardrop) but are also subject to variation due to changes in the position of limbs and pelvis [6].

This method has however been reported to be as reliable as plain radiological scanograms [7] and reproducible, with a measurement error of ±1 mm [5]. There is presently no accepted guideline or body of evidence with the aim to standardise practice for obtaining pre-operative radiographs for the purpose of accurate templating for THA. In this regard, a standard of imaging would be invaluable to ensure optimal conditions for the surgeon to perform THA in the pre-operative setting. 

## Materials and methods

A retrospective, single-centre study consisting of 213 consecutive patients who underwent elective THA for osteoarthritis during a two-year period was performed. Patients were identified from a prospectively collected adult hip arthroplasty database in a dedicated UK arthroplasty unit. Inclusion criteria were adult patients undergoing planned primary THA for hip osteoarthritis. Exclusion criteria included patients with missing data, spinal deformity/misalignment and hip flexion deformity. Thus, after exclusions, the radiographs of 195 patients were analysed. This was deemed an adequate sample size for the outcome to be sufficiently powered at 80%. All radiographs were stored and reviewed on the Picture Archiving and Communication System (PACS; Agfa HealthCare, Mortsel, Belgium). 

Radiographic assessment

The quality of radiographs was assessed by two senior authors. Assessors were blinded to the details of the patients, and any cases they were involved in were excluded. Two radiographs were assessed for each patient: (1) AP pelvic radiograph immediately before the operation and (2) AP pelvic radiograph immediately after the operation. Each radiograph was assessed on the following criteria devised by the authors to provide adequate exposure and a non-rotated anatomical image: 

- Patient position and alignment were rated as good, fair or poor, respectively, depending upon whether ≥2, at least 1 or none of the following were met. 

- PS and coccyx are positioned in a straight line in the middle of the screen with 1-3 cm between the superior edge of the PS and the tip of the coccyx. 

- Greater (GT) and lesser (LT) trochanters are clearly distinguishable. 

- Obturator rings are symmetric.

The orientation of the X-ray beam was assessed by measuring the visible area above the iliac crest (perpendicular distance from the top of the iliac crest (if included) to the proximal-most point of the radiograph), the visible length of the proximal femur (from the tip of the GT to the distal-most point included in the radiograph), the shortest distance from the centre point of the radiograph to the PS and the uppermost vertebral body visible. All post-operative images were also assessed on whether the distal tip of the implanted prosthesis was visible or not. The total number of radiographs taken for each patient at the pre-operative and post-operative stages was also recorded. 

Statistical analysis 

All statistical analyses were performed using SPSS version 26.0 (IBM, Armonk, NY). Continuous variables were summarised as mean (standard deviation [SD], minimum and maximum values) or median (interquartile range [IQR; 25th-75th percentile], minimum and maximum values) as appropriate. Categorical variables were summarised by frequency and percentage. Chi-squared analysis (with Fisher’s exact test) was used to evaluate differences in categorical data. A p-value threshold of 0.05 was used for statistical significance. 

## Results

Of the 213 patients who met the inclusion criteria, 18 were excluded. The sample consisted of 195 patients; the mean age was 66 years SD ± 14 (range 25-92 years); 123 (63%) were female. All included patients had pre- and post-operative images assessed. Only 23.6% of the post-operative radiographs met the criteria to be categorised as ‘good’ compared with 59% of the pre-operative radiographs (p < 0.001). A significantly greater proportion of the post-operative radiographs were categorised as either ‘fair’ (58.4%) or ‘poor’ (15.4%) compared to the pre-operative radiographs (‘fair’ [36.4%; p < 0.001] and ‘poor’ [4.6%; p < 0.001], respectively) (Table 1).

**Table 1:** Summary of the radiographic quality assessment

	Good	Fair	Poor
Pre-operative radiographs (n = 195)	115 (59%)	71 (36.4%)	9 (4.6%)
Post-operative radiographs (n = 195)	46 (23.6%)	114 (58.4%)	30 (15.4%)
	X^2^ = 47.8; p < 0.001	X^2^ = 21.45; p < 0.001	X^2^ = 12.56; p < 0.001

**Figure 1:**
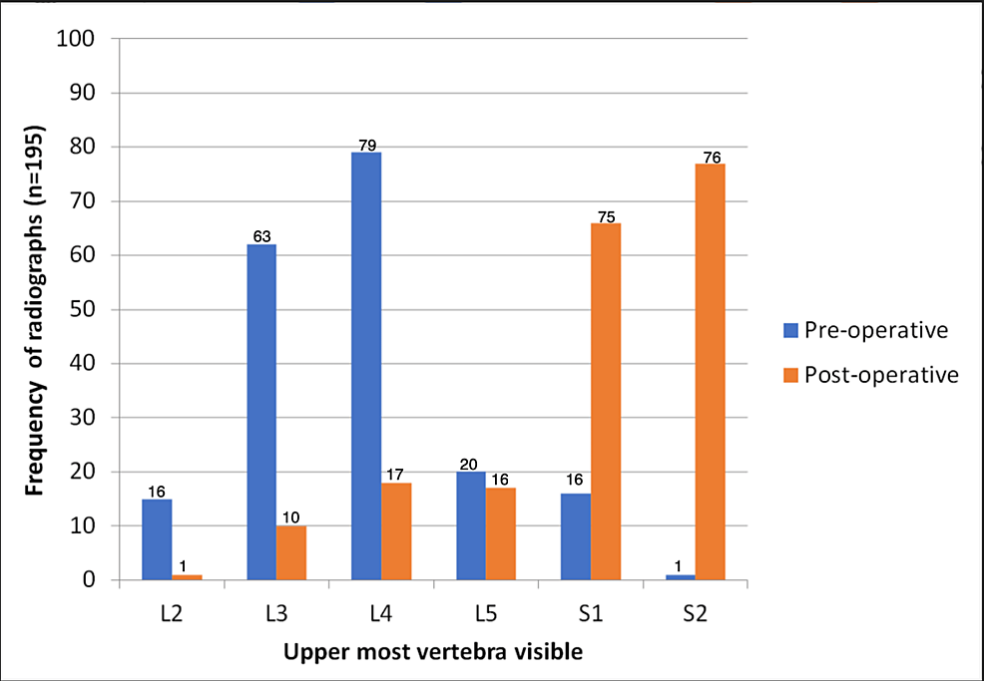
Comparison of the uppermost vertebral level visible on pre- and post-operative radiographs Blue = pre-operative radiographs; orange = post-operative radiographs.

**Figure 2:**
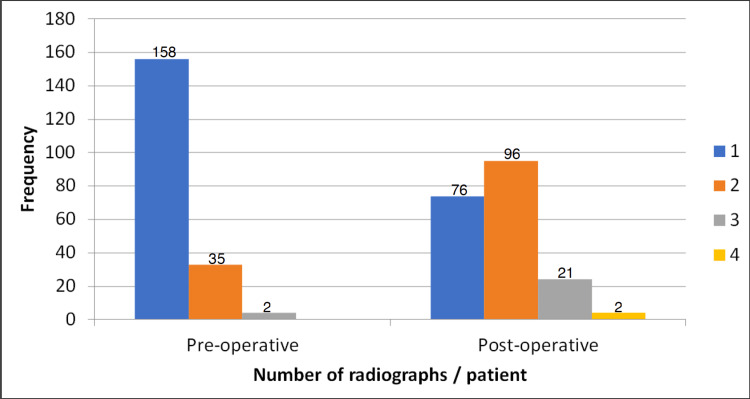
Comparison of the total number of radiographs taken for each patient at the pre- and post-operative stages Each colour represents the total number of radiographs taken.

The centre points of the pre-operative radiographs were above the PS (mean distance +34 mm) while for post-operative radiographs the centre points were below the PS (mean distance -26 mm), p < 0.001. Consequently, 80% of the pre-operative radiographs showed L4 or higher vertebras whereas 80% of the post-operative radiographs showed L5 or lower vertebral bodies (Figure 1). The mean perpendicular distance above the iliac crests was 30.6 mm in the pre-operative radiographs compared to 4.9 mm in the post-operative radiographs (p < 0.001). The mean length of the proximal femur visible in the pre-operative radiographs was 177 mm compared to 220 mm in the post-operative radiographs (p < 0.001). Although more of the femur was visible in the post-operative radiographs, 25.6% of them did not show the distal tip of the implanted prosthesis. The total number of radiographs taken for each patient was higher in the post-operative stage (median 2, [IQR 1]), compared with the pre-operative stage (median 1 [IQR 0]) (Figure 2). 

## Discussion

An ageing population has resulted in an increase in the number of elective hip arthroplasty procedures being performed globally. Therefore, there is a clear need to investigate methods of optimising surgical outcomes. It is known that good pre-operative planning for THA by use of templating delivers significant benefits with regard to selecting implant size and position, allows for contingency planning as well as improves intra-operative efficiency [8]. Studies highlight the continuing importance of pelvic radiographs for this [3] and naturally, the quality of radiographs on which the template is based must be adequate. A recent review by Di Martino et al. (2023) highlights the importance of using good-quality pelvic radiographs in enabling adequate templating and surgical planning to optimise implant performance [9]. This review highlights how pelvic radiographs should be used to determine the hip rotation centre and femoral offset during acetabular templating. Different methods exist to calculate this including Fisher’s method and Ranawat’s method. This forms the basis for femoral templating to achieve the correct alignment and thus femoral offset and limb length. Standardisation of pelvic radiographs is necessary to allow these calculations and although this review describes important qualitative factors, currently no guidelines exist to ensure the quality of pelvic radiographs prior to and after THA [10]. This study highlights that a standardised protocol to ensure the quality of pre-operative pelvic radiographs is needed to ensure accurate templating and the benefits this entails. 

One of the intra-operative challenges of THA is correcting LLD without affecting the stability of the hip joint. Pre-existing LLDs should be accounted for during pre-operative planning and corrected at the time of THA [11,12]. A common complication of THA is post-operative limb lengthening [9,13], which may have been caused due to hip trial prosthesis instability. This may have been managed with methods to increase femoral offset and implant height, risking iatrogenic limb lengthening [14]. 

Plain radiographs are routinely used to assess various key factors to improve or maintain in THA including LLD for example [15]. It is difficult to ascertain an LLD that is acceptable to patients. However, studies have shown that there can be a range between 3 and 17 mm in LLD [16]. The consequences of an LLD can be quite catastrophic for patients where even a perceived LLD can lead to poor patient-reported outcomes. It has been associated with back pain, sciatica, dislocation, and early loosening of implants [16]. It can be seen in previous studies that less than 50% of patients who had perceived LLD were not concerned with this complication. However, 31% of patients required the use of a shoe raise as a method of adjusting LLD and avoiding revision. Whilst the perception of patients' LLD was high, radiologically only 36% of patients had an anatomical LLD [17]. Naturally, minimising LLD by appropriate radiological templating pre-operatively would help optimise patient satisfaction post-operatively.

The radiographic assessment showed only 59% of pre-operative imaging, and 23.6% of post-operative imaging was deemed good in terms of quality of assessment for THA. Even though the majority of pre-operative radiographs included the L4 vertebrae providing adequate exposure of the iliac crests, they did not meet other criteria to be classified as good quality, suggesting adjustments to magnification may be necessary. Pre-operative imaging is essential in the initial templating process. A recent systematic review showed that pre-operative templating significantly increased the accuracy of a THA, including providing a good estimate of the overall success of the procedure and decreasing post-operative complications [18]. This further stresses the need for gold-standard guidelines related to pre- and post-operative imaging. Standard AP pelvic radiographs can no longer be considered an adequate imaging technique for THA templating as these radiographs typically centre on the sacrum and thus the third proximal femur may not be adequately imaged with this method. It is recommended that pelvic radiographs have the X-ray beam centred on the PS to visualise the proximal femur sufficiently [19]. The quality of such radiographs pre- and post-operatively using a scoring criterion that evaluates factors that are recognised as essential in THA radiographs was assessed. We must also consider that variation in imaging quality may arise from a lack of universally accepted standards in literature. The only published criteria for quality assessment of pelvic radiographs were provided by the Commission of European Communities (CEC) in 1996. However, important criteria are missing (e.g. inclusion of iliac crests in AP pelvis radiographs) [20]. Despite an extensive literature search, no validated visual grading scale for image quality assessment of AP pelvis images to be used for THA templating has been identified. This further enhances the view that a standardised guideline is essential in the future management of patients requiring a THR. We suggest the following criteria should be met for adequate pre-operative radiographs enabling accurate templating by providing adequate exposure and symmetrical anatomical imaging: 

- The patient should be lying supine with the hips in 10° to 15° of internal rotation. 

- The X-ray beam should be centred on the distal end of the PS. 

- PS and coccyx should be in a straight line in the middle of the screen with 3 cm (in men) and 4 cm (in women) between the superior edge of the PS and the tip of the coccyx. 

- Greater (GT) and lesser (LT) trochanters should be clearly distinguishable and symmetric. 

- Obturator rings should be symmetric. 

- The proximal third of the femur should be visible. 

Post-operatively, radiographs are taken to confirm the acceptable positioning of the prosthesis. Varus or valgus mispositioning of the prosthesis can be identified by visualising the distal tip of the prosthesis. In the post-operative radiographs, 25.6% did not show the distal tip of the prosthesis. Furthermore, the majority of patients had at least two radiographs taken post-operatively to ensure the prosthesis was imaged appropriately. This increases the radiation burden to the patient and the time taken by the radiographer. Ideally, the entire prosthesis should be clearly visible in one image and clear guidelines should be outlined and followed post-operatively to achieve this.

Limitations to this study include the fact that due to retrospective data collection, we were unable to assess if the quality of pre-operative imaging had an effect on surgical outcomes. The value of accurate pre-operative templating is evident in the literature where calculation of femoral offset and leg-length discrepancies enables appropriate implant positioning and sizing increasing patient satisfaction and implant longevity whilst reducing post-operative complications [18]. Assessing surgical outcomes was out of the scope of this study, which highlights the high proportion of ‘inadequate’ radiographs due to the lack of a protocol. Accurate templating on 2D radiographs and the consequential benefits are only possible if the radiographs are of a high standard. Thus, a protocol with specific criteria is suggested using available literature to this end. Furthermore, the number of radiographs taken may not be representative of the total number of images taken as all may not have been saved. However, the fact that repeat imaging was needed multiple times post-operatively suggests that they were often inadequate. Additionally, although we appreciate that templating using 3D images such as CT and EOS® imaging is valuable in deformed hips, those with excessive degenerative changes and those undergoing revision surgery, good-quality plain radiographs remain adequate for the majority of cases [3]. 

## Conclusions

Standard pelvic radiographs are not adequate for appropriate pre-operative planning for hip arthroplasty (centred at the sacrum, upper level at L3 with little femur visible). 

We recommend a specific pelvic radiograph protocol for hip arthroplasty, which is centred on the PS at the lesser trochanter level to adequately cover the area above the acetabulum and maximum length of the femur allowing appropriate pre-operative planning.
